# Revisiting cholesterol metabolism in hepatocellular carcinoma: a hidden driver of systemic therapy response

**DOI:** 10.1016/j.esmogo.2025.100284

**Published:** 2026-02-13

**Authors:** L.M. Stoffels, E.S. Espinoza Rodriguez, J. Theys, R. Shiri-Sverdlov

**Affiliations:** 1 Department of Precision Medicine, Institute for Oncology & Reproduction (GROW), Maastricht University, Maastricht, The Netherlands; 2 Department of Genetics and Cell Biology, Research Institute of Nutrition and Translational Research in Metabolism (NUTRIM), Maastricht University, Maastricht, The Netherlands

**Keywords:** hepatocellular carcinoma, cholesterol, systemic therapy

## Abstract

Hepatocellular carcinoma (HCC) ranks as the fourth leading cause of cancer-related mortality worldwide and poses a substantial challenge in the field of oncology. The majority of patients are diagnosed at advanced stages of the disease, where systemic therapies remain the primary treatment modality. These interventions, however, are frequently constrained by limited efficacy and considerable adverse effects, highlighting the pressing need for more precise and effective therapeutic strategies. Emerging evidence has identified cholesterol as a critical factor in HCC pathogenesis, influencing key processes such as tumor initiation, cellular proliferation, immune dysregulation, and metastatic progression. Moreover, disruptions in cholesterol metabolism have been increasingly implicated in resistance to systemic treatments. This review provides a concise overview of therapeutic approaches targeting cholesterol in HCC and examines the influence of cholesterol metabolism on the efficacy of systemic therapies, particularly those currently considered standard of care. Finally, we discuss existing challenges and propose future directions for integrating cholesterol-lowering strategies to enhance treatment outcomes in patients with HCC.

## Highlights

• Cholesterol plays a critical role in HCC pathogenesis by promoting tumor initiation, proliferation, and progression.
• Emerging preclinical data suggest a role for cholesterol metabolism disruption in systemic therapy resistance.
• Modulating cholesterol represents a promising strategy with potential to alter the course of HCC.
• Further studies are needed to validate the potential of cholesterol modulation to enhance systemic therapy efficacy in HCC.

## Introduction

Hepatocellular carcinoma (HCC) is the most common form of primary liver cancer, accounting for ∼80% of all cases.^1^ It poses a significant global health burden, ranking as the fourth leading cause of cancer-related mortality and the second-leading cause of cancer-related years of life lost worldwide.^2^ Major risk factors include chronic infection with hepatitis B or C virus, chronic alcohol consumption, and metabolic dysfunction-associated steatotic liver disease (MASLD).^3^

The management of HCC is highly dependent on disease stage. In early-stage HCC, curative options including liver transplantation and hepatic resection provide the best long-term survival outcomes.^1^^,^^4^ The majority of patients, however, are diagnosed at advanced stages, where systemic therapies remain the primary treatment approach.^5^ Sorafenib, a multikinase inhibitor, has long been the standard first-line therapy for advanced HCC, after which lenvatinib emerged as effective alternative.^6^^,^^7^ More recently, the combination of the immune checkpoint inhibitor (ICI) atezolizumab with the anti-vascular endothelial growth factor (VEGF) antibody bevacizumab has demonstrated superior overall survival (OS) benefits compared with sorafenib.^8^ Despite these advances, treatment resistance, contributing to disease recurrence and limited OS, remains a significant obstacle, highlighting the need for novel therapeutic strategies.

Cholesterol has emerged as a critical contributor to HCC pathogenesis.^9^ Beyond its fundamental role in maintaining cell membrane integrity, cholesterol functions as a precursor for bile acids, steroid hormones, and vitamin D. It is derived from dietary sources and endogenous synthesis, with its metabolism tightly regulated by key enzymes, such as 3-hydroxy-3-methylglutaryl coenzyme A (HMG-CoA) reductase. Dysregulation of cholesterol uptake and metabolism, however, can have significant pathological consequences.^10^ Several preclinical studies have underscored the impact of dietary cholesterol on the general onset of HCC and development of HCC from MASLD.^11^, ^12^, ^13^ One key mechanism by which cholesterol contributes to aggressive proliferation of HCC cells is by serving as a critical metabolic fuel sustaining their rapid growth. This dependency is further supported by the up-regulation of cholesterol biosynthesis regulators, such as squalene epoxidase (SQLE) and sterol regulatory element-binding protein 2 (SREBP2), which are frequently elevated in HCC tumor tissues and contribute to enhanced proliferation in HCC cells.^14^, ^15^, ^16^ Beyond promoting proliferation, preclinical evidence suggests that cholesterol overload can contribute to apoptotic resistance, and invasion and metastasis in HCC.^17^, ^18^, ^19^ Notably, in addition to the role of cholesterol in the initiation and progression of HCC, accumulating evidence points towards its key role in determining the efficacy of systemic therapies.

Given its central role in tumor biology, cholesterol metabolism represents a promising target for therapeutic intervention to enhance outcome in HCC. In this review, we will first discuss emerging cholesterol-targeting therapeutic strategies and their implications for HCC. Furthermore, we will explore the impact of cholesterol on systemic therapy outcomes in HCC, focusing on its metabolic regulation, immunological interactions and potential contribution to treatment efficacy. Lastly, we will discuss clinical implications and future directions of cholesterol-modulating therapies in HCC.

## Potential modalities to target cholesterol in HCC

Cholesterol is intricately linked to the pathogenesis of HCC, with disruptions in its uptake and biosynthesis frequently observed in the disease. Therefore, implementing cholesterol-lowering drugs may offer clinical benefits in HCC. In this section, we describe the potential modalities for targeting cholesterol in HCC and the preclinical evidence supporting their therapeutic potential.

Statins, widely used as cholesterol-lowering agents, inhibit HMG-CoA reductase, the rate-controlling enzyme of the mevalonate pathway responsible for cholesterol synthesis. Preclinical studies show that statin use could suppress the development of hepatic tumors and reduce HCC growth in murine models.^20^, ^21^, ^22^, ^23^ Notably, elevated concentrations of HMG-CoA reductase in tumor tissue correlates with poorer survival outcomes in HCC patients.^24^ Early clinical evidence further supports the potential of statins in reducing HCC risk and improving outcomes in patients. A case-control study showed that statin use was associated with reduced risk of HCC.^25^ Furthermore, the use of pravastatin has been associated with prolonged survival in patients with advanced HCC in a randomized, controlled trial.^26^ Collectively, these findings highlight the potential of statins in both the prevention and treatment of HCC.

Disturbed cholesterol balance/metabolism can also be targeted using liver X receptor (LXR) agonists which function as sterol sensors protecting cells from cholesterol accumulation by inducing reverse cholesterol transport and activation of its conversion into bile salts. He et al.^27^ showed that treatment with the LXR agonist T317 reduced migration and colony formation in liver cancer cells, and reduced development of xenograft Huh7 tumors in mice. Similarly, Rudalska et al.^28^ showed that LXR agonist treatment was well tolerated in mouse models for metabolic dysfunction-associated steatohepatitis (MASH)-HCC and xenografted HCC, and overcame therapy resistance. Despite these promising results, some LXR agonists have also been reported to raise triglyceride concentrations in preclinical studies, requiring further evaluation as high triglyceride concentrations can increase the risk for health conditions including heart disease and pancreatitis.^29^, ^30^, ^31^, ^32^

Farnesoid X receptor (FXR) agonists represent another category of cholesterol-targeting agents with potential application for HCC. FXR is a critical regulator of normal cholesterol homeostasis by controlling bile acid synthesis, and activation of FXR improves insulin sensitivity and lowers blood cholesterol. Preclinical evidence suggests that FXR agonists could decrease HCC growth and metastasis.^33^^,^^34^ FXR agonists may also help prevent fibrosis-driven hepatocarcinogenesis, as obeticholic acid reversed fibrosis in a toxic cirrhosis rat model.^35^

Proprotein convertase subtilisin/kexin type 9 (PCSK9) inhibitors have also been investigated in HCC for their cholesterol-lowering capabilities. PSCK9 acts via binding to low-density lipoprotein receptors (LDLR) on hepatocytes leading to their internalization and degradation within lysosomes, resulting in higher plasma cholesterol concentrations. High expression of PSCK9 in tumor tissues has been shown to be related to large tumor size in HCC patients and associated with poor prognosis.^17^^,^^36^ Pharmacological inhibition of PCSK9 reduced cell growth and migration in Huh7 and HepG2 cells, and reduced aggressiveness in a chorioallantoic membrane model.^37^^,^^38^ Inhibition of PCSK9 has also been shown to alleviate inflammation and fibrosis via AMP-activated protein kinase/mechanistic target of rapamycin/Unc-51-like kinase 1 (AMPK/mTOR/ULK1) signaling in a mouse model for liver fibrosis.^39^ While PCSK9 inhibitors have been well studied in cardiovascular diseases, their potential in HCC requires further research.

Moreover, cyclodextrins, cyclic oligosaccharides which contains a hydrophobic cavity allowing transport of cholesterol out of the cell, have been explored as potential therapeutic strategies. Although studies in HCC are currently lacking, preclinical evidence in models for breast cancer suggest that cholesterol modulation by hydroxypropyl-β-cyclodextrin (HP-β-CD) inhibited proliferation and metastasis.^40^ Moreover, in a mouse model for MASLD, cyclodextrin treatment reduced intracellular cholesterol concentrations.^41^ Given its promising effects in preclinical studies, future research should explore its applicability in HCC.

Also, natural compounds with cholesterol-lowering properties may hold potential for HCC. Plant stanols and sterols, also known as phytosterols, are natural compounds found in plants that can interfere with cholesterol absorption in the gut. Studies suggest that diets rich in phytosterols may reduce the risk of cancer.^42^ Other natural compounds with cholesterol-lowering properties and potential anti-HCC effects include for instance emodin, a bioactive anthraquinone. Emodin has been shown to lower cholesterol in rat models for hyperlipidemia and hypercholesteremia, and promising data have been obtained in a mouse model for HCC.^43^, ^44^, ^45^

Overall, modulating cholesterol, both through pharmacological and natural compounds, represents a promising strategy with potential to alter the course of HCC.

## Impact of cholesterol on systemic treatment efficacy

Given its important role in the onset of HCC and progression, an increasing number of studies are examining how cholesterol affects treatment outcomes (Table 1). Such evidence allows for better understanding of cholesterol-mediated treatment resistance and its potential targeting to enhance sensitivity. This section describes the impact of cholesterol on treatment, focusing on the mainstay systemic treatments for HCC.

**Table 1:** Targets of cholesterol regulation that impact systemic therapy

Target	Key findings/implications		Study type	References
	Sorafenib/lenvatinib	ICI blockade		
SREBP2	Higher SREBP2 expression correlates with shorter DFS in sorafenib-treated HCC.	HCC tumors express higher concentrations of SREBP2 and PD-L1	HCC samples	Mok et al. (2022)^46^; Shao et al. (2025)^47^
	Targeting SREBP2 enhances sorafenib/lenvatinib efficacy		Preclinical	Fan et al. (2023)^16^; Kim et al. (2018)^45^
SREBF2		Inhibiting SREBPF2 transcription with PD-1 blockade reduces tumor growth	Preclinical	Liu et al. (2025)^48^
SPARC	Up-regulated in HCC tumors with lower sorafenib sensitivity		HCC samples	Wan et al. (2024)^49^
ABCB1	Activation of ABCB1 facilitated lenvatinib exocytosis		*In vitro*	Hu et al. (2022)^50^
SCAP	Targeting SCAP (i.e. lycorine) with sorafenib inhibits tumor growth	ZDHH3 inhibitor reduces SCAP acetylation, enhancing anti-PD-1 therapy	Preclinical	Li et al. (2022)^51^; Wu et al. (2024)^52^
HMG-CoA	Lovastatin enhances efficacy of sorafenib/lenvatinib	Atorvastatin enhanced response to PD-1 blockade	Preclinical	Shao et al. (2022)^53^; Shwe et al. (2021)^54^
	Statins with sorafenib/lenvatinib improves OS and PFS		Retrospective study	Han et al. (2024)^55^
	Pravastatin did not improve OS when combined with sorafenib		Clinical study	Jouve et al. (2019)^56^; Riaño et al. (2020)^57^
		Atorvastatin could reduce PD-L1 expression in HepG2 cells	*In vitro*	Shwe et al. (2021)^54^
		Nanodelivery of simvastatin synergized with PD-L1 therapy	Preclinical	Yu et al. (2022)^58^
STARD4	STARD4 knockdown enhances lenvatinib efficacy		Preclinical	Liu et al. (2025)^59^
NPC2	Reduction of expression of NPC2 diminishes sorafenib efficacy		*In vitro*	Suk et al. (2021)^60^
SQLE		Targeting SQLE restored effectiveness of anti-PD-1	Preclinical	Wen et al. (2024)^61^; Qiao et al. (2025)^62^
PCSK9		Inhibition of PCSK9 improved anti-PD1 immunotherapy	Preclinical	Xu et al. (2024)^63^

ABCB1, ATP-binding cassette B1; DFS, disease-free survival; HCC, hepatocellular carcinoma; HMG-CoA, 3-hydroxy-3-methyl-glutaryl coenzyme A; ICI, immune checkpoint inhibitor; NPC2, Niemann-Pick intracellular cholesterol transporter 2; OS, overall survival; PCSK9, proprotein convertase subtilisin/kexin type 9; PD-1, programmed cell death protein 1; PD-L1, programmed death-ligand 1; PFS, progression-free survival; SCAP, SREBP cleavage-activating protein; SPARC, secreted protein acidic and rich in cysteine; SQLE, squalene epoxidase; SREBF2, sterol regulatory element-binding transcription factor 2; SREBP2, sterol regulatory element-binding protein 2; STARD4, StAR-related lipid transfer protein 4.

### Cholesterol induces resistance to tyrosine kinase inhibitors in HCC

Recent preclinical studies have highlighted the interaction of cholesterol and genes related to cholesterol biosynthesis on the efficacy of sorafenib and lenvatinib, tyrosine kinase inhibitors (TKI) used in the treatment of HCC.^49^,^64^ Notably, there is convincing evidence that sorafenib- and lenvatinib-resistant cells exhibit higher concentrations of SREBP2, compared with sorafenib-sensitive cells.^46^ SREBP2 is a transcription factor that primarily regulates cholesterol biosynthesis by controlling the expression of genes involved in cholesterol and lipid metabolism. Elevated SREBP2 activity, and consequently increased cholesterol biosynthesis, has been shown to contribute to sorafenib resistance by inducing sonic hedgehog (SHH) signaling.^46^ SHH signaling is critical for the self-renewal of neoplastic cells, including cancer stem cells (CSCs).^65^ CSCs are widely recognized as key contributors to treatment resistance through various mechanisms, including their capacity to induce cell cycle arrest, self-renew, and differentiate into heterogenous cancer cell lineages.^66^ Clinical analysis of HCC tissues from patients treated with sorafenib further showed that high expression of SREBP2 in HCC samples correlated with shorter disease-free survival.^46^ Targeting SREBP2 has shown promise in overcoming resistance. Emodin, a bioactive compound derived from traditional Chinese herbs, has been shown to inhibit SREBP2 transcriptional activity, thereby suppressing cholesterol biosynthesis and protein kinase B (Akt) signaling, and enhancing the antitumor efficacy of sorafenib *in vivo*.^45^ The Akt pathway plays a central role in mediating multidrug resistance, including inhibition of apoptosis, rendering survival signals, and facilitating cancer stemness.^67^ Similarly, Fan et al.^16^ demonstrated elevated SREBP2 expression in HCC tissue compared with non-HCC. Inhibition of SREBP2 using betulin, a natural extract from bark of birch trees, enhanced the antitumor effect of lenvatinib by suppressing the mTOR/IL-1β pathway.^16^ Similarly, StAR-related lipid transfer protein 4 (STARD4), a soluble protein having a critical role in intracellular cholesterol transport, was increased in tumor tissue of HCC patients, and its knockdown *in vivo* enhanced lenvatinib sensitivity.^59^

In addition to SREPB2, secreted protein acidic and rich in cysteine (SPARC) is an important player in cholesterol biosynthesis and has been shown to be up-regulated in HCC tumors with lower sorafenib sensitivity.^49^ Mechanistic studies revealed that sorafenib-resistant HepG2 cells contained more membrane cholesterol, and that SPARC up-regulation could induce sorafenib resistance in a cholesterol-dependent manner.^49^ Moreover, membranes rich in cholesterol are known to contain and stabilize ATP-binding cassette (ABC) transporters, such as P-glycoprotein (P-gp), which facilitate drug efflux.^68^ Supporting this, Hu et al.^50^ demonstrated that lenvatinib resistance is linked to disrupted cholesterol metabolism and lipid raft formation. Specifically, activation of ABCB1 by EGFR in a lipid raft-dependent manner facilitated enhanced exocytosis of lenvatinib in HCC cells.^50^

Beyond cholesterol accumulation in the plasma membrane, cholesterol buildup in mitochondria can also impair sorafenib sensitivity. Sterol regulatory element binding transcription factor 2 (SREBF2) has been shown to up-regulate STARD4, thereby facilitating mitochondrial cholesterol trafficking and decreasing mitochondrial membrane potential, leading to reduced cytochrome C release.^64^ Normally, cytochrome C release into the cytoplasm triggers the intrinsic apoptotic pathway, and higher concentrations of cytochrome C are associated with a favorable prognosis.^69^ When cytochrome C release is diminished due to cholesterol buildup in the mitochondria, however, cancer cells evade apoptosis.^69^^,^^70^ Knockdown of SREBF2 has been shown to reverse mitochondrial cholesterol accumulation and restore sorafenib sensitivity in HCC mouse models.^64^ In line, data based on the Cancer Genome Atlas show that a prognostic model based on mitochondrial cholesterol metabolism can predict the response to sorafenib and immunotherapy of HCC patients.^71^

Cholesterol can potentially affect the efficacy of TKIs in HCC by altering autophagy. Li et al.^51^ demonstrated that SREBP cleavage-activating protein (SCAP) was increased in sorafenib-resistant tissues and cells. SCAP regulates cholesterol biosynthesis by inducing translocation of SREBP. Knockdown of SCAP increased the numbers of autophagosomes and enhanced AMPK activity. Moreover, targeting SCAP using lycorine combined with sorafenib inhibited tumor growth in a preclinical model of HCC.^51^ Shao et al.^53^ further emphasized the impact of cholesterol on autophagy and efficacy of TKIs. Specifically, cholesterol accumulation reduced autophagy of receptor tyrosine kinases (RTK) in a Golgi-membrane protein 1-dependent manner. Cholesterol reduction using lovastatin enhanced the efficacy of sorafenib, lenvatinib, and regorafenib in a subcutaneous preclinical model for HCC by inducing RTK autophagy.^53^

Disruption of lysosomal cholesterol transport has been shown to impact sorafenib efficacy. Lower expression of Niemann-Pick type C2, resulting in free cholesterol accumulation in lysosomes, diminished sorafenib efficacy in HCC cells by enhancing MAPK/AKT signaling. Mice on a high-cholesterol diet were demonstrated to have increased tumor growth, which could be attenuated by the cholesterol-lowering agent HP-β-CD used to treat Niemann-Pick disease type C.^60^

Limited clinical data are available on combining cholesterol-lowering strategies with TKIs, mostly focused on statins. Han et al.^55^ demonstrated that co-administration of statins with sorafenib or lenvatinib improved OS and progression-free survival (PFS) in patients with advanced HCC. Notably, patients who continued or initiated statin use after TKI treatment had better OS, whereas discontinuation of statin therapy correlated with poorer OS.^55^ Conversely, randomized, controlled trials have reported conflicting results. Jouve et al.^56^ reported no significant OS improvement when sorafenib was combined with pravastatin compared with sorafenib alone in advanced HCC patients. Similarly, another clinical trial showed that while the combination of sorafenib and pravastatin prolonged time to progression, it had no effect on OS.^57^ Importantly, these clinical trials focused solely on pravastatin, leaving the potential benefits of other statins and other cholesterol-lowering modalities unexplored.

Taken together, multiple preclinical studies suggest that increased cholesterol contributes to TKI resistance and highlight the potential of targeting cholesterol to improve sensitivity although clinical evidence remains controversial.

### Cholesterol affects efficacy of immune checkpoint inhibitors in HCC

Emerging evidence highlights the role of dysregulated cholesterol not only in the efficacy of TKI but also in impacting ICI therapy. Despite improved response rates with first-line treatments such as atezolizumab and bevacizumab, overall efficacy remains suboptimal, with evidence suggesting that dysregulated cholesterol may impair their effectiveness through its impact on the tumor microenvironment (TME) and T-cell modulation.^72^^,^^73^

The TME of HCC is typically marked by an accumulation of exhausted T cells, which limits the effectiveness of the immune response.^74^ Cholesterol plays a significant role in shaping this immunosuppressive environment. Studies have shown that HCC tissues enriched in cholesterol induces cytotoxic T-cell exhaustion, marked by up-regulation of ICI receptors such as programmed death-ligand 1 (PD-1), lymphocyte-activating gene-3 (LAG-3), and 2B4.^75^^,^^76^ This exhausted phenotype is marked by decreased production of critical effector molecules, including granzyme B, interferon-γ (IFN-γ) and tumor necrosis factor-α (TNF-α), in a dose-dependent manner.^75^ Consistent with these findings, Shao et al.^47^ reported higher protein concentrations of both SREBP2 and PD-L1 in HCC tumor tissues compared with control. They also found that cholesterol inhibits the ubiquitination and degradation of PD-L1 in HCC cells.^47^ Notably, atorvastatin could reduce the protein expression of PD-L1 induced by IFN-γ or IFN-γ/TNF-α in HepG2 cells.^54^ The relationship between cholesterol and PD-L1 appears to be bidirectional. While cholesterol up-regulates PD-L1 expression, PD-L1 itself has been implicated in lipid reprogramming within tumors. Overexpression of PD-L1 promoted accumulation of triglycerides, cholesterol, and lipid droplets in hepatoma cells, as demonstrated in both *in vitro* and *in vivo* studies.^77^ Notably, further evidence suggests that maintaining a delicate balance in cholesterol metabolism is essential to preserve effective T-cell function. Specifically, while elevated cholesterol can dampen immune responses, low cholesterol concentrations have been shown to impair T-cell proliferation and induce autophagy-mediated apoptosis. This impairment is driven by dysregulation of key regulatory factors such as SREBP2 and LXR, which reduce cholesterol acquisition while enhancing cholesterol efflux, further impairing T-cell functionality.^78^

Beyond its direct impact on T cells, cholesterol may impair the efficacy of ICI therapy in HCC by perpetuating a pro-inflammatory state.^79^ Dysregulated cholesterol metabolism is intricately linked to chronic inflammation, a key driver of hepatocarcinogenesis. In turn, chronic inflammation exacerbates ICI resistance and induces PD-L1 expression in HCC cells, establishing a self-reinforcing cycle that undermines immunotherapy efficacy.^54^^,^^80^ Li et al.^81^ reported that inhibition of the SREBP pathway in hepatocytes reduced HCC progression by reduction of expression of tumor-promoting cytokines. Complementing this, He et al.^82^ described the vicious cycle formed by cholesterol accumulation and nuclear factor-κB (NF-κB) activation in the presence of lipopolysaccharides, which significantly up-regulated the expression of HMG-CoA reductase, LDLR, and SREBP2, while reducing PCSK9 expression in liver cancer cells. Cholesterol may also impact ICI efficacy by impacting antigen presentation ability of dendritic cells. Elevated cholesterol concentrations within dendritic cells impair their migration ability and present tumor antigens effectively.^83^ Villablanca et al.^84^ showed that human and mouse tumors released cholesterol metabolites leading to LXR activation thereby inhibiting migration of dendritic cell and T-cell priming, ultimately facilitating immune escape of tumors. As a result, weakened antigen presentation reduces T-cell activation and proliferation, undermining the effectiveness of immunotherapies that rely on antigen presenting cell–T cell interactions to elicit antitumor responses.

Several preclinical studies have investigated the potential of targeting cholesterol metabolism to enhance therapeutic outcome of ICIs in HCC. For instance, Wen et al.^61^ demonstrated that SQLE facilitates cholesterol accumulation within the TME, which impairs effector T-cell function. Inhibiting SQLE restored the effectiveness of anti-PD-1 treatment in models for MASH-associated HCC, where anti-PD-1 alone had no impact on tumor growth. This observation was further confirmed in a preclinical study for HCC showing that targeting SQLE synergized with anti-PD1 therapy. Importantly, SQLE expression was associated with poorer response to ICB in HCC patients.^62^ Cholesterol metabolism also modulated immune response through additional pathways. High expression of C-X-C motif chemokine ligand 2 (CXCL2) in HCC patients, which suppress cholesterol biosynthesis via SREBF2, was associated with improved response to anti-PD-1 treatment, and overexpression improved PD-1 blockade *in vivo*.^48^ In addition, PCSK9 has been linked to poorer overall survival and reduced markers of CD8+ T cells. Pharmacological inhibition of PCSK9 improved the anti-HCC efficacy of anti-PD1 immunotherapy *in vivo*, suppressing tumor growth in a CD8+ T cell-dependent manner.^63^ Similarly, a ZDHH3 inhibitor to reduce SCAP/SREBP2 signaling and tumor cholesterol synthesis, reduced HCC growth in mice treated with anti-PD-1.^52^ Statins further support therapeutic relevance: Shao et al.^47^ demonstrated that lowering cholesterol concentrations with lovastatin enhanced the response to PD-1 blockade in a xenograft HCC model, underscoring the potential of statins in improving ICI outcome. Similarly, Yu et al.^58^ developed a nanodelivery system for simvastatin targeting liver sinusoidal endothelial cells, which synergized with PD-L1 antibody therapy in a murine model of advanced HCC. Mechanistically, this targeted reduction in cholesterol synthesis promoted recruitment of natural killer cells in fibrotic tissue and up-regulation of the activation markers CD69 and IFN-γ.^58^

Although clinical studies investigating the role of cholesterol in immunotherapy for HCC remain limited, some evidence highlights its significance. Lu and Lu^85^ identified total cholesterol concentrations as predictive marker for PFS (according to the RECIST 1.1 score) and OS in patients receiving a combination of ICI and TKIs for advanced HCC. Similarly, Li et al.^86^ demonstrated that HCC patients with high serum cholesterol concentrations had shorter PFS and OS, along with lower response rates to anti-PD-1 therapy, whereas patients with low serum cholesterol concentrations demonstrated better response. It should be noted, however, that these studies are associative, and OS may be determined by comorbidities influenced by elevated serum cholesterol rather than a causal effect on tumor biology. Supporting this trend, statin use has been associated with improved responses to PD-L1 antibody therapy in lung cancer, raising the possibility that similar benefits could extend to HCC patients undergoing immunotherapy.^87^

**Figure 1:**
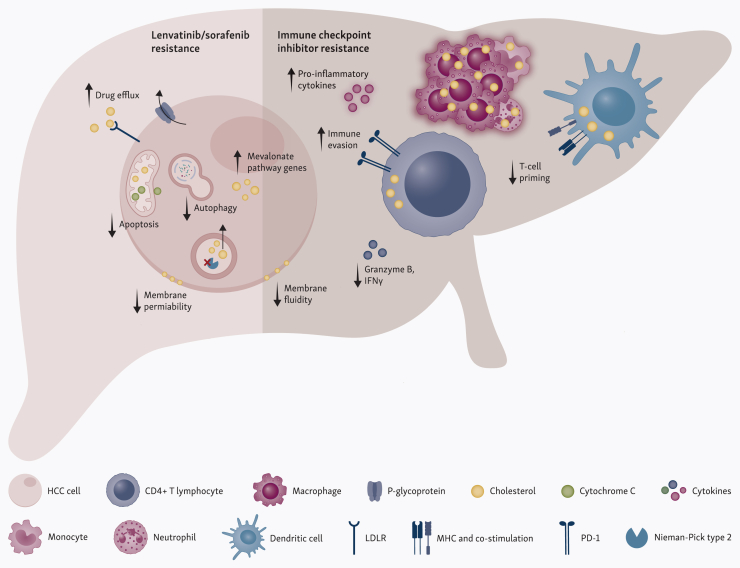
**Cholesterol modulates the response to systemic therapies in HCC.** The accumulation of cholesterol, driven by increased synthesis and uptake, may reduce the efficacy lenvatinib and sorafenib by promoting drug efflux and disrupting mitochondrial and lysosomal function, among other mechanisms (left panel). In parallel, dysregulated cholesterol metabolism can impair T-cell function and antigen presentation, while promoting a pro-inflammatory tumor microenvironment that compromises the efficacy of immune checkpoint inhibitors (right panel). HCC, hepatocellular carcinoma; LDLR, low-density lipoprotein receptor; MHC, major histocompatibility complex; PD-1, programmed cell death protein 1. Created with BioRender.com.

Overall, evidence suggests a critical role for cholesterol in modulating the TME, influencing T cells and immune responses. Targeting cholesterol metabolic pathways in combination with immunotherapy may enhance therapeutic efficacy, though studies in a clinical setting are required to further validate this (Figure 1).

## Challenges and future directions

Unlike diseases that are characterized by hepatic lipid disturbances (e.g. MASH), in which increased serum cholesterol and hepatic cholesterol accumulation are often linked, in cancer this correlation is less clear as cancer cells often demonstrate disturbed cholesterol biosynthesis and consumption.^88^ So far, potential strategies for cholesterol modulation and the impact of cholesterol on systemic therapy has been demonstrated predominantly in preclinical studies. Implementing cholesterol-modulating approaches in clinical practice may offer significant clinical benefits when incorporated as adjuvant therapies, although several challenges remain. One such challenge is that cholesterol-lowering interventions may not benefit all patients. HCC is a highly heterogeneous disease, often arising in the context of underlying liver dysfunction, such as cirrhosis or chronic hepatitis, adding a layer of complexity to treatment strategies. For instance, the safety of statin therapy in patients with underlying liver conditions remains a subject of debate.^89^ While numerous studies highlight the benefits of statins in reducing the incidence and progression of MASLD, their use is contraindicated in patients with chronic liver disease or decompensated cirrhosis, due to safety concerns.^90^ The delicate interplay between cholesterol metabolism, tumor biology, and liver function underscores the need for a more nuanced, personalized approach.

In addition, further investigations to examine whether cholesterol-lowering therapies differently affect systemic therapy response across different etiologies for HCC are warranted. While lipid-lowering therapies, specifically statins, have been linked to decreased HCC risk both in viral^91^^,^^92^ and MASLD-associated liver disease,^93^ evidence specifically examining their impact on systemic therapy outcomes in HCC is, to our knowledge, lacking. Current evidence suggests that efficacy of TKIs in HCC is not affected by etiology.^94^ In contrast, the effect of etiology on immunotherapy response is less clear: although some evidence suggest MASH-HCC responds differently to HCC, there is no consensus to support a differential response among etiologies.^94^^,^^95^ Considering the central role of metabolic overload and concomitant oxidative stress and inflammation in MASH, an important avenue for future research includes evaluating whether cholesterol modulation differentially affects immunotherapy response in etiology.

Importantly, lowering cholesterol is not always beneficial as cholesterol plays a critical role in cellular functions, including immune regulation. Excessive cholesterol reduction can lead to dysfunctional immune responses, potentially limiting antitumor immunity.^78^^,^^96^ For instance, low cholesterol in T cells could inhibit their proliferation, thereby impairing functionality.^78^ Thus, striking the right balance is crucial, as both high and low cholesterol concentrations may have unintended consequences in HCC patients.

Another critical barrier to clinical translation is the current lack of robust clinical data. To establish a clear therapeutic role for cholesterol-lowering strategies, well-designed clinical intervention studies are needed. Currently, no clinical trials have systematically investigated the impact of cholesterol-lowering interventions with immunotherapy in HCC. Importantly, these studies should extend beyond statins and evaluate alternative cholesterol-targeting, including FXR agonists, cyclodextrins, and non-pharmacological interventions. A broader, more inclusive approach to clinical investigation will be essential to fully understand the therapeutic potential of cholesterol modulation in enhancing systemic treatment efficacy for HCC.

Extending this area of research to encompass other treatment modalities, such as resection of radiotherapy ablation, may also be valuable as emerging evidence suggests that cholesterol-lowering therapies could offer benefits beyond systemic therapies. For instance, one study demonstrated the ability of statins to lower post-surgical recurrence rates of HCC over follow-up periods of 1 to 5 years.^97^ Similarly, combined treatment of transarterial chemoembolization (TACE) with pravastatin improved survival of advanced HCC patients compared with TACE alone in a prospective cohort.^98^ Moreover, although not in the context of HCC, preclinical studies suggest that radioresistant cancer cells, including pancreatic and colorectal cancer cells, have higher expression of genes involved in cholesterol synthesis.^99^^,^^100^ In line, Geyer et al.^101^ showed that high total cholesterol was associated with decreased OS in both patients with early and advanced HCC, undergoing radiofrequency ablation alone, combined with sorafenib, or selective internal radiation thereby. Thus, while most preclinical studies to date have focused on how cholesterol influences systemic therapy of HCC, it may be worthwhile to expand research into its potential interactions with other therapeutic approaches. Understanding how statins or other cholesterol-lowering interventions interact with these therapies could provide valuable insights into optimizing comprehensive HCC treatment strategies.

## Conclusion

Overall, cholesterol modulation presents a promising strategy to improve response to systemic therapy and overall outcome in HCC. Given the heterogeneity of HCC, cholesterol-targeting therapies should not be viewed as a universal solution but rather as part of a personalized treatment approach. Future studies should focus on both mechanistic insights and clinical validation to determine the true potential of cholesterol modulation in HCC management.
